# Long-Lasting Consequences of Neonatal Maternal Separation on Social Behaviors in Ovariectomized Female Mice

**DOI:** 10.1371/journal.pone.0033028

**Published:** 2012-03-07

**Authors:** Mumeko C. Tsuda, Sonoko Ogawa

**Affiliations:** Laboratory of Behavioral Neuroendocrinology, Graduate School of Comprehensive Human Sciences, University of Tsukuba, Tsukuba, Ibaraki, Japan; University of New England, Australia, Australia

## Abstract

Maternal separation (MS) stress is known to induce long-lasting alterations in emotional and anxiety-related behaviors, but effects on social behaviors are not well defined. The present study examined MS effects on female social behaviors in the social investigation (SIT) and social preference (SPT) tests, in addition to non-social behaviors in the open-field (OFT) and light-dark transition (LDT) tests in C57BL/6J mice. All females were tested as ovariectomized to eliminate confounding effects of endogenous estrogen during behavioral testing. Daily MS (3 hr) from postnatal day 1 to 14 did not affect anxiety levels in LDT, but were elevated in OFT with modified behavioral responses to the novel environment. Furthermore, MS altered social investigative behaviors and preference patterns toward unfamiliar stimulus mice in SIT and short- and long-term SPT paradigms. In SIT, MS reduced social investigation duration and increased number of stretched approaches towards both female and male unfamiliar stimulus mice, suggesting increased social anxiety levels in MS females. Similarly, MS heightened levels of social anxiety during short-term SPT but no MS effect on social preference was found. On the other hand, MS females displayed a distinctive preference for female stimuli, unlike control females, when tested for long-term SPT over a prolonged period of 5 days. Evaluation of FosB expression in the paraventricular nucleus, medial and central amygdala following stimulus exposure demonstrated greater number of FosB immunopositive cells in all three brain regions in MS females compared to control females. These results suggest that MS females might differ in neuroendocrine responses toward unfamiliar female and male opponents, which may be associated with modifications in social behaviors found in the present study. Taken together, this study provides new evidence that early life stress modifies female social behaviors by highlighting alterations in behavioral responses to situations involving social as well as non-social novelty.

## Introduction

There is growing evidence that childhood exposure to adverse experiences can increase the vulnerability to develop psychopathologies related to emotional and social disturbances later in life [1], [2]. This suggests that the early life period is a sensitive age in which stressful events can adversely affect brain development and subsequent behavioral phenotypes. Maternal separation (MS) is a well-utilized animal model that mimics the stress of early negative life experiences by separating pups from their mother daily for several hours during their first 2 weeks of life. This forced absence of the dam in MS rats and mice consequently produces long-lasting alterations in neuroendocrine, cognitive, and behavioral functions that persist into adulthood [3]–[5].

A large number of studies have reported MS effects in rats, but in recent years, few studies have demonstrated MS-induced alterations in emotionality and anxiety levels in both sexes of mice [6], [7]. However, behavioral changes expressed in MS mice remain inconsistent, particularly in females. In female mice, daily MS of 3 hours from postnatal day 1 to 14 has been reported to increase, decrease, or not alter levels of emotionality and anxiety [6], [8], [9]. It should be noted that females in these studies were tested as gonadally intact, and the confounding effects of estrogen at the time of testing were not taken into account. It is known that estrogen modulates emotionality and anxiety levels in non-social and social contexts in female mice in a dose-dependent manner [10]. Recently, Romeo et al. showed that effects of MS on emotionality and anxiety levels were detected in female mice tested in diestrus (when estrogen levels were low), but not in females tested in estrus (when estrogen levels are high) [8]. This suggests that fluctuating levels of endogenous estrogen may be a confounding factor in MS effects on emotionality and anxiety-related behaviors. Therefore, we found it essential to test female mice as ovariectomized to eliminate the influence of fluctuating estrogens on emotionality and anxiety levels measured in both non-social and social contexts in the present study.

To date, effects of MS on female social behaviors are largely unknown. In humans, childhood traumatic events are known to be associated with an increased risk of developing social anxiety disorder and social phobia in adulthood and interestingly, such risks are found to be higher in women than in men [2], [11], [12]. In mice, various types of social interaction tests based on the natural tendency for mice to investigate a stranger mouse [13] have been used to evaluate social behavioral deficits [14]. Unfortunately, animal models studying the effects of early life stress on adult female social behaviors are still lacking. Thus, the present study investigated the effects of MS on female social behaviors in a newly developed social behavior paradigm in our laboratory, the social investigation test (SIT). In SIT, similar to what we have previously reported [10], experimental mice are exposed to sensory (visual, auditory, and olfactory) cues, but prevented from any direct physical contact to an intruder mouse in their home territory. By testing experimental mice in this given condition, behavioral responses to social stimuli could be assessed without the influential factors of novelty of the testing apparatus and stress caused by the presentation of an intruder mouse. With the use of SIT, we examined whether MS altered social investigative behaviors toward an unfamiliar opponent mouse in adult female mice and if MS-induced alterations depend on whether the stimulus mouse is female or male.

Furthermore, stressful situations are reported to alter the female's preference for female and male stimuli [15]–[18]. Specifically, acute stress can decrease the female's preference for a male opponent in preference tests. To elucidate whether MS alters the female's social preference for female and male opponents, the present study utilized two newly developed types of social preference tests (SPT) in our laboratory, one that is a short-term version while the other a long-term version. In rodents, higher levels of social anxiety can disrupt the preference between two stimuli mice in a preference test [19]. To determine if social preference of females subjected to MS are affected by social anxiety, we examined whether exposure time to stimuli mice influences preference patterns in short-term (15 min) or long-term (5 days) SPT. Additionally, studies in humans reveal that early stress experience can heighten the risk of developing social phobia disorders [12], [20], [21]. Therefore, we also assessed whether MS modified the female's propensity to spend time with another mouse. Taken together, with the use of both SIT and SPT behavioral tests, we aimed to gain insight into how early life stress affects female social behavior towards unfamiliar individuals in adulthood.

Additionally, in the present study, we aimed to understand possible underlying brain processes involved in MS-induced alterations in female social behaviors, particularly during initial social interactions. Therefore, we evaluated neuronal activation following social stimulus exposure using FosB as a marker. The expression of FosB, a member of the Fos family of transcription factors that also includes c-fos, has been used as a marker for cell activation in response to a number of stimuli [22], [23]. After acute stimuli, FosB is rapidly induced (as early as one hour), but can also persist in the brain for longer periods due to their stability [24]. Here, we examined changes in the induction of FosB expression one hour following a 15 min exposure to an unfamiliar stimulus mouse in the paraventricular nucleus of the hypothalamus (PVN), and the central (CeA) and medial amygdala (MeA), which are brain regions associated with the regulation of social behaviors and stress responses.

In summary, the present study aimed to investigate the effects of neonatal MS stress not only on non-social, but also on social behaviors in C57BL/6J ovariectomized female mice.

## Methods

### Ethics Statement

All experimental and surgical procedures were approved by the University of Tsukuba Animal Care and Use Committee (permit numbers 08–043, 09–027, 10–017) and were strictly conducted in accordance with the National Institutes of Health guidelines. All efforts were made to minimize suffering.

### Animals

Adult nulliparous female and male C57BL/6J mice purchased from CLEA (Tokyo, Japan) were mated. During their last week of gestation, pregnant females were individually housed in plastic cages (29×19×12 cm) with cotton nesting material and monitored daily for parturition. Day of parturition was defined as postnatal day (PND) 0. All mice were maintained on a 12∶12 light/dark cycle (lights off at 1200) and at a constant temperature (23±2°C) throughout the study. Food and water were provided *ad libitum*.

### Maternal Separation Procedure

Each litter was culled to six pups (female: male ratio as close to 1∶1 as possible) and randomly assigned to either MS or non-separated control groups on PND 1. In the MS group, pups were separated daily for 3 hr from the dam between 1500 (3 hr after lights off) and 1800 from PND 1 to 14. The dam was first removed from her home-cage and placed in a separate cage with clean bedding, which was transferred to a separate room during the entire 3 hr separation period. All pups were then removed from their nest, placed together in a small container filled with clean bedding and kept on a warmer maintained at 36°C. After the 3 hr separation, all pups were returned to their home-cage, immediately followed by the dam. For the control group, dams and pups remained together between 1500 and 1800.

Between PND 1 and 14, all home-cages were changed twice and during each cage change, a small amount of bedding from the old home-cage was transferred and mixed with fresh bedding of the new home-cage. On PND 21, all pups were weaned and group-housed with littermates of the same sex. Animals were then left undisturbed until testing in adulthood, except for routine animal care procedures that included weekly cage changes. Only female offspring mice were used in the present study.

### Experimental Groups

At 12 wk of age, female offspring from both treatment groups were bilaterally ovariectomized (OVX) under anesthesia with isoflurane inhalation (Dainippon Sumitomo Pharma, Japan) and individually housed at this time. Control and MS females were then assigned to one of the four behavioral testing groups that examined the effects of MS on (1) emotional and anxiety-related behaviors in OFT and LDT, (2a) social investigative behaviors towards an unfamiliar female or male opponent mouse in SIT followed by (2b) social preference patterns for either female, male, or absence of stimuli over a long-term 5 day exposure period in SPT, (3) social preference for female or male stimuli mice during a short-term 15 min exposure time in SPT, or (4) FosB expression following exposure to an unfamiliar female or male stimulus mouse.

### Measurement of Emotional and Anxiety-Related Behaviors

#### Mice

At 13 wk of age, emotionality and anxiety-related behaviors of control (n = 12) and MS (n = 23) OVX female mice were first examined in OFT and then LDT two days later. OFT was conducted under low intensity light conditions to examine emotional behavioral responses as well as general activity levels in an unfamiliar environment, while in LDT, anxiety-related behaviors were measured relatively independent of exploratory activity levels. All tests were conducted during the dark phase of the light/dark cycle starting 2 hr after lights off.

#### Open Field Test (OFT)

Mice were tested for 10 min in an open field apparatus (60×60×30 cm) illuminated with low intensity lighting (5 lux) to examine behavioral responses in a novel, unfamiliar situation. The floor of the apparatus was hypothetically divided into 25 square sections (16 outer and 9 inner). At the beginning of each test, mice were placed in the same corner with its head facing the corner. Activity were monitored and analyzed on a Macintosh computer using Image OFC 2.03 (O'Hara & Co., Ltd), modified software based on the public domain NIH Image program (developed at the U.S. National Institute of Health and available on the internet at http://rsb.info.nih.gov/nih-image/). Total moving distance, time spent in center area, and vertical activity (rearing postures without leaning on the wall of open field apparatus) was recorded for each mouse. Upon completion of test, mice were returned to its home cage and the apparatus was thoroughly wiped clean.

#### Light-Dark Transition Test (LDT)

Test apparatus consisted of an enclosed black dark compartment (0 lux) and an open-top, brightly lit white compartment (350 lux), each with dimensions of 40×20×25 cm. A small doorway (5×2 cm) between compartments allowed mice to move freely between compartments. At the beginning of each test, mice were placed into the dark compartment and the doorway automatically opened 5 sec later. Horizontal activity and time spent in each compartment was recorded for 10 min on a Windows computer using Image J LD2 (O'Hara & Co., Ltd), modified software based on the public domain Image J program (see above). Cumulative time spent in the light compartment, latency to enter the light compartment, and number of transitions between the two compartments was then calculated as indices of anxiety-related behaviors. At the end of each test, mice were returned to their home-cage and the apparatus was thoroughly wiped clean.

### Measurement of Social Behavioral Responses Toward Unfamiliar Stimulus Mice

#### Mice

At 13 wk of age, OVX control (n = 29) and MS (n = 30) female mice were examined once for social behavioral responses in their home territory toward either a female or male stimulus mouse in SIT. All tests were conducted during the dark phase of the light/dark cycle starting 2 hr after lights off.

#### Social Investigation Test (SIT)

Testing apparatus (SOSI TYPE1, O'Hara & Co., Ltd., Fig. S1A) consisted of a white plastic testing cage placed centrally in a white polyvinyl chloride box (46×51×25 cm) and the entire set-up was illuminated with white lighting (26 lux). Forty-eight hours before testing, mice were transferred from their housing cage to testing cage (31×35×17 cm, Fig. S1B) with clean bedding and allowed to establish home territory. Prior to testing, mice were acclimated to an empty cylinder (see below) placed in the center of their cage for 30 min. Then, females were tested for behavioral responses toward either a cylinder containing an unfamiliar group-housed OVX female (10 control and 13 MS) or single-housed gonad ally intact male (15 control and 16 MS) C57BL/6J mouse for 15 min. All stimuli mice were obtained from the breeding colony maintained at the University of Tsukuba and were 13–20 wk old at the time of testing. Stimulus mice were placed in clear Plexiglas cylinders (7 cm in diameter at the bottom and 4.4 cm in diameter at the top, 16 cm in height) consisting of 28 holes (6 mm in diameter) near the bottom 3 cm of the cylinder (Mouse Cylinder SIOT1, O'Hara & Co., Ltd., Fig. S1C) and introduced into the center of the females' cage. These holes allowed the experimental mouse to be exposed to olfactory cues from the stimulus mouse, but prevented from any direct physical contact between the two mice except the tip of their nose. All cylinders were thoroughly washed, wiped with 70% ethanol, and then air-dried. Throughout testing, test cages were covered with a clear acrylic board.

Behavior during SIT was videotaped with a camera placed 39 cm above the testing cage (Fig. S1A). Video recordings were scored by an experimenter not aware of the identity of the mice using a digital event recorder program (Recordia 1.0b, O'Hara & Co., Ltd.) for the measurements of social investigation time (cumulative duration of sniffing towards perforated parts of the cylinder; Fig. S1D), duration of sniffing from corner (mouse is in a sitting position in one of the corners of the cage, fixes its head in the direction of the cylinder and sniffs the air; Fig. S1E) and number of stretched approaches (total number of stretches, in which the mouse extends its head and forefeet toward the cylinder while tightly holding their hind legs at the original position; Fig. S1F).

### Measurement of Social Preference for Female and Male Stimuli Mice

#### Mice

The same OVX control (n = 29) and MS (n = 30) female mice used in SIT were tested for long-term SPT at 16 wk of age (3 wk after SIT) in one of three stimuli sets, (1) female vs. male mouse, (2) female vs. no stimulus mouse, and (3) male vs. no stimulus mouse. Since these experimental female mice were previously tested in SIT against either a female or male stimulus mouse, SPT stimuli sets presented to females were appropriately counterbalanced. Specifically, experimental female mice tested against a female stimuli in SIT were equally distributed between the three stimuli set groups for SPT and experimental mice tested against a male stimuli in SIT were treated similarly. A separate set of control (n = 19) and MS (n = 15) OVX female mice were tested for social preference between an unfamiliar female and male stimulus mouse in a short-term SPT at 14 wk of age.

#### Short-Term Social Preference Test (SPT)

Testing apparatus, cages, and cylinders used were the same as those described in SIT. Forty-eight hours before testing, mice were transferred to a white plastic testing cage with clean bedding and allowed to establish home territory. Before testing, experimental mice were acclimated to two empty cylinders placed in two corners of their cage for 30 min. Female mice were then tested for preference between an unfamiliar, group-housed OVX female and single-housed, gonadally intact male C57BL/6J mice for 15 min. All stimulus mice were obtained from the breeding colony maintained at the University of Tsukuba and were 13–20 wk old at the time of testing. They were placed in clear Plexiglas cylinders and introduced into the same two corners of the experimental mouse's cage used during acclimation. All tests were conducted during the dark phase of the light/dark cycle starting 2 hr after lights off.

During testing, behavior of experimental mice was videotaped and scored by an experimenter using the Recordia digital event recorder program for measurements of social investigation time (cumulative duration of sniffing towards perforated parts of the cylinder) of each stimulus-containing cylinder.

#### Long-Term Social Preference Test (SPT)

Experimental mice were tested for a total of eight days. The apparatus (AMAZENG TYPE1, O'Hara & Co., Ltd., Fig. S2) used in this experiment consisted of one large plastic cage (20×30×14 cm) connected to two small cages (28×11×11 cm) by a clear acrylic tunnel (7×6×6 cm). At the end of each tunnel, wire mesh with 1 cm diameter openings was inserted between the small cage and tunnel to prevent transitions between the large and small cages, as well as physical contact between experimental and stimulus mice except the tip of their nose. Each tunnel was equipped with an infrared beam, which recorded the cumulative time spent in each tunnel by the experimental mouse housed in the large cage every 15 min on a Windows computer using the Time BAP software (O'Hara & Co., Ltd). All three cages were covered with wire metal tops and provided with food and water *ad libitum*. All mice were maintained on a 12∶12 light/dark cycle (lights off at 1200) and at a constant temperature (23±2°C).

On the first day of testing, experimental mice were placed in the large cage and acclimated to the apparatus with two empty small cages for the next two days. On the third day, stimuli mice were introduced into the two small cages and social preference of experimental mice was continuously assessed for the next 5 days (testing period). The fifty-nine female mice were assigned to one of the following stimuli sets, (1) OVX female vs. gonadally intact male mouse (8 control and 11 MS), (2) OVX female mouse vs. empty cage (10 control and 10 MS), or (3) gonadally intact male mouse vs. empty cage (11 control and 9 MS). Stimuli mice were group-housed OVX ICR/Jcl females and single-housed gonadally intact ICR/Jcl males obtained from the ICR/Jcl breeding colony maintained at the University of Tsukuba. For each experimental mouse, the cumulative time spent in each tunnel connected to stimuli containing cages were measured during the testing period. Furthermore, daily preference percentage ([time spent in the tunnel connected to the cage presented with stimuli *X*/total time spent in both tunnels]*100) during the 5 day testing period was also calculated.

### Measurement of FosB Expression Following Stimulus Mice Exposure

#### Mice

At 14 wk of age, twenty-four C57BL/6J female mice were examined for FosB expression following the exposure to an unfamiliar female (4 control and 4 MS) or male (4 control and 4 MS) stimulus mouse, or for basal measurements of FosB (4 control and 4 MS).

#### Stimulus Mice Exposure

Experimental female mice were exposed to either a cylinder (see SIT methods) containing an unfamiliar group-housed OVX female or single-housed gonadally intact male C57BL/6J mouse once for 15 min in the SIT set-up. All stimuli mice were obtained from the breeding colony maintained at the University of Tsukuba and were 13–20 wk old at the time of testing. During exposure, behaviors were video-recorded and cumulative duration of social investigation and number of stretched approaches toward the stimulus-containing cylinder were measured.

#### Immunohistochemistry

One hour after initial exposure to stimulus mice, mice were deeply anesthetized with an intraperitoneal injection of a 1∶1 solution containing sodium pentobarbital (60 mg/kg, Kyoritsu Seiyaku Co., Japan) and sodium heparin (1000 units/kg, Nipro Pharma Co., Japan). They were then perfused transcardially with 0.1 M phosphate-buffered saline, pH 7.2, followed by 4% paraformaldehyde (PFA) in 0.1 M phosphate buffer (PB), pH 7.2. Brains were removed, postfixed in 4% PFA in PB, immersed in 30% sucrose in PB for 48 hr at 4°C and coronally sectioned at 30 µm on a freezing microtome. Tissues from mice not exposed to stimulus mice were also processed in the same manner for measurements of basal levels of FosB expression.

Free-floating sections were washed in 1% hydrogen peroxide in 0.05 M tris-buffered saline (TBS; pH 7.2) containing 0.2% triton X-100 (TBS-X) for 20 min to inhibit endogenous peroxidase activity, thoroughly washed several times in TBS-X, and then blocked in an incubation buffer containing 3% normal goat serum (NGS; Vector Laboratories, USA) and 3% bovine serum albumin (BSA; Sigma-Aldrich, USA) in TBS-X for 2 hr at room temperature. Free-floating sections were then incubated in 1) a rabbit polyclonal FosB antiserum (0.2 mg/ml, sc-48, Santa Cruz, USA) dissolved in the incubation buffer overnight at 4°C, 2) a 1∶250 dilution of biotinylated goat anti-rabbit secondary antibody (Vector Laboratories, USA) dissolved in the incubation buffer for 2 hr at room temperature, and 3) avidin-biotin-complex (Vectastain ABC Elite kit, Vector Laboratories, USA) in TBS for 1 hr at room temperature. Sections were visualized with 0.03% 3,3′-diaminobenzidine (DAB) containing 0.15% nickel ammonium sulfate and 0.03% hydrogen peroxide in TBS. Then, sections were mounted on gelatin-coated slides, air-dried, dehydrated through ascending alcohol series, which were cleared with xylene, and coverslipped with Permount (Fisher Scientific, USA).

Six anatomically matched sections containing PVN (Bregma −0.58 to −1.22 mm), CeA (Bregma −1.06 to −1.70 mm) and MeA (Bregma −1.22 to −1.82 mm) were obtained and each brain area was photographed at 4× magnification with a digital camera mounted on an Olympus microscope (BX61, Olympus, Japan). FosB immunopositive cells were bilaterally counted by an experimenter unaware to the experimental conditions of the animal using the Adobe Photoshop Creative Suite software (Adobe Systems Inc., USA). Total number of positive cells was then calculated in each brain region for each animal for further statistical analysis.

### Statistical Analysis

OFT and LDT data were analyzed by a one-way ANOVA for treatment differences. During SIT, four control females and one MS female data were excluded from analysis due to no activity during the 20 min SIT testing. The remaining SIT behavioral data and number of FosB immunopositive cells in each brain region were analyzed with a two-way ANOVA for main effects of treatment, stimuli, and their interactions. Short-term and long-term SPT data were analyzed with either a one-way ANOVA for treatment differences of total time spent with each stimulus or by a paired t-test to compare the differences in time spent investigating between paired stimulus mice. Furthermore, the daily preference percentage calculated for long-term SPT was analyzed by two-way ANOVA for repeated measurements for main effects of treatment, day, and their interactions in each stimuli set group. When appropriate, ANOVAs were followed by a Bonferroni post hoc test and significant differences were considered when *p*<0.05. All data was analyzed using SPSS 14.0J (SPSS Inc., Chicago, IL) statistical package.

## Results

Since two or more offspring from each litter were tested in OFT, LDT, and short-term SPT, behavioral data were first analyzed by a one-way ANOVA with litter as an independent variable in each treatment group to exclude the possibility of litter effects. No statistically significant effect of litter was found in any behavioral parameters measured in OFT, LDT, and short-term SPT (Table S1).

### MS Effects on Emotional and Anxiety-Related Behaviors

#### Open-Field Test

**Figure 1:**
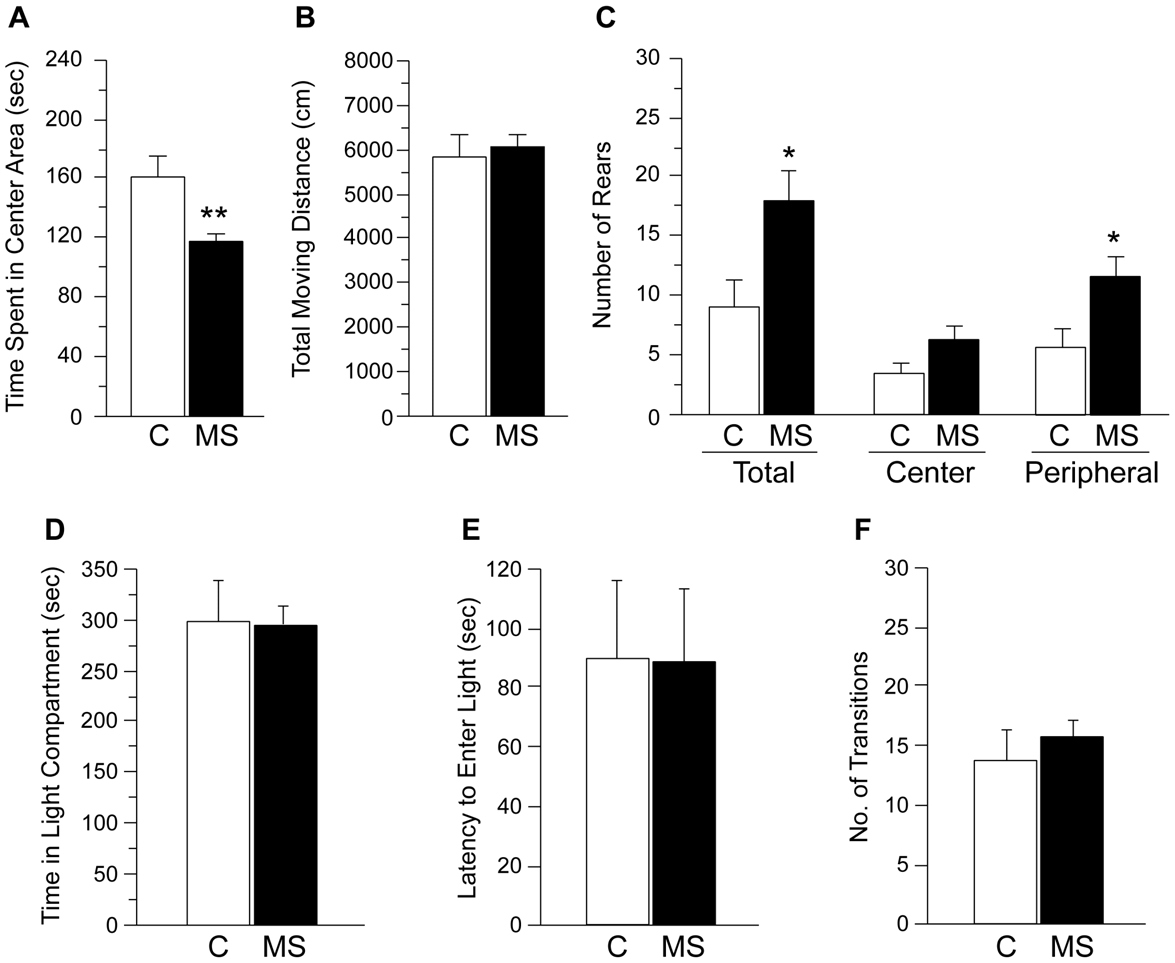
MS effects on anxiety-related behaviors in OFT and LDT. (A) The cumulative time spent in the center area, (B) total moving distance in the whole open field arena, and (C) number of rears within the whole open field arena, center area of the arena, and peripheral area of the arena during OFT were measured in control and MS female mice. No effect of MS was observed in the behavioral measurements of (D) time spent in the light compartment, (E) latency to enter the light compartment, and (F) number of transitions between compartments in female mice during LDT. All data are presented as mean + SEM. *, *p*<0.05; **, *p*<0.01 vs. control. C: control; MS: maternal separation.

MS significantly affected behaviors measured in OFT (Fig. 1A–C). Specifically, compared to control females, MS females spent less time in the center area ([F (1,33) = 9.95, *p*<0.01], Fig. 1A), although total moving distance did not differ (Fig. 1B). Furthermore, MS females displayed higher amounts of total rears ([F (1,33) = 5.60, *p*<0.05], Fig. 1C) compared to control mice. Detailed analysis of rearing within the open field arena revealed that compared to control group, MS females showed a greater number of rears in the peripheral area ([F (1,33) = 5.68, *p*<0.05]; Fig. 1C), but not in the center area. Taken together, these results suggest modified behavioral responses to a novel, non-social environment in MS female mice.

#### Light-Dark Transition Test

No effect of MS was observed in anxiety-related behaviors as measured by the time spent in the light compartment ([F (1,33) = 0.01, *p* = 0.93]; Fig. 1D). Additionally, no statistically significant differences between treatment groups were found in the latency to enter the light compartment ([F (1,33) = 0.08, *p* = 0.98]; Fig. 1E) and number of transitions between the two compartments ([F (1,33) = 0.36, *p* = 0.55]; Fig. 1F).

### MS Effects on Social Investigative Behaviors

**Figure 2:**
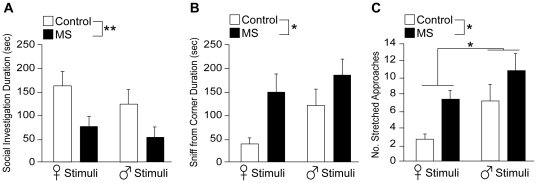
MS-induced reduction in social investigative behaviors toward unfamiliar female and male stimulus mice during SIT. (A) Cumulative social investigation duration, (B) total sniffing from corner duration, and (C) number of stretched approaches toward either a female or male stimulus-containing cylinder. All data are presented as mean + SEM. *, *p*<0.05; **, *p*<0.01.

MS greatly modified social behavioral responses towards both female and male unfamiliar stimuli mice. Social investigation time was significantly shorter in MS females compared to control females regardless of the sex of stimulus mouse ([treatment: F (1,50) = 9.47, *p*<0.01, stimuli: F (1,50) = 1.46, *p* = 0.23, treatment × stimuli: F (1,50) = 0.09, *p* = 0.77]; Fig. 2A and Video S1 and S2). On the other hand, overall duration of sniffing from corner ([treatment: F (1,50) = 6.16, *p*<0.05, stimuli: F (1,50) = 2.79, *p* = 0.10, treatment × stimuli: F (1,50) = 0.40, *p* = 0.53]; Fig. 2B) and number of stretched approaches ([treatment: F (1,50) = 5.73, *p*<0.05, stimuli: F (1,50) = 5.01, *p*<0.05, treatment × stimuli: F (1,50) = 0.10, *p* = 0.76]; Fig. 2C) was significantly increased in MS females compared to control females. Additionally, the number of stretched approaches was significantly higher towards male stimuli mice, regardless of treatment (*p*<0.05; Fig. 2C). Altogether, these results suggest heightened levels of social anxiety toward unfamiliar stimuli mice in MS females compared to the control group.

### MS Effects on Social Preference Patterns

#### Short-Term Social Preference

**Figure 3:**
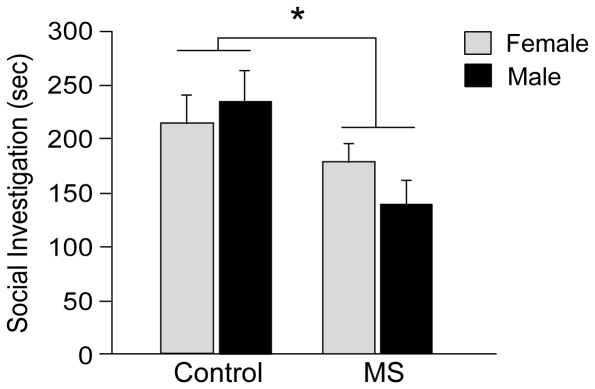
MS effects on social preference for female and male stimuli mice during short-term SPT. MS females displayed reduced levels of total social investigation time towards both stimulus-containing cylinders in female mice. In addition, control females did not show preferences for either stimulus, whereas MS females tended to spend less time towards the male stimulus mouse. All data are presented as mean + SEM. *, *p*<0.05. MS: maternal separation.

In both MS and control female mice, no statistically significant preference between male and female stimuli mice was found, although MS females may have tended to spend more time with the female rather than male stimulus mouse ([t (14) = 2.06, *p* = 0.06]; Fig. 3). It should be noted that consistent with SIT results, total duration of social investigation was significantly lower in MS females compared to control female mice [F (1,32) = 5.954, *p*<0.05] suggesting heightened anxiety levels in MS females in short-term SPT.

#### Long-Term Social Preference

**Figure 4:**
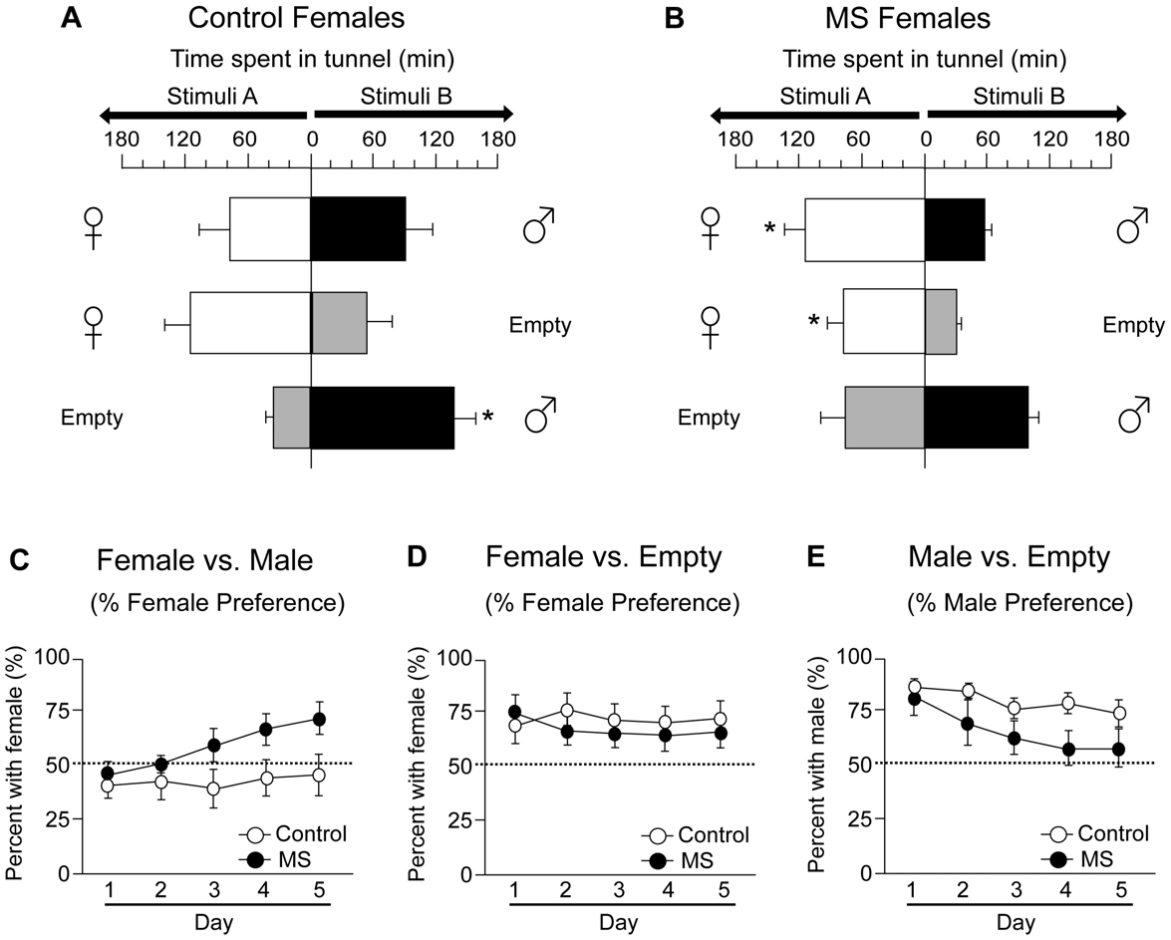
MS-induced alterations in social preference during either ‘female vs. male,’ ‘female vs. empty,’ or ‘male vs. empty’ stimuli sets during long-term SPT. Total time spent in each tunnel connected to stimuli cages, indicated as length of horizontal bars, was measured in each stimuli paradigm during the dark phase in (A) control and (B) MS female mice. Female and male symbols on the left and right columns represent stimulus animals. Empty indicates the absence of a stimulus animal in the stimuli cage. Daily preference percentages during the 5 testing days within each stimuli paradigm in control and MS females are shown in C–E. (C) Percent of female stimuli preference in ‘female vs. male’ stimuli set, (D) percent of female stimuli preference in ‘female vs. empty cage’ stimuli set, and (E) percent of male stimuli preference in ‘male vs. empty cage’ stimuli set on each testing day. All data are presented as mean + SEM. MS: maternal separation.

The daily cumulative duration female mice spent in each of the two tunnels during the 12 hr dark phase were averaged over the 5 day testing period and shown in Fig. 4A (control mice) and Fig. 4B (MS mice). Although total duration, as indicated as the length of the horizontal bars, did not significantly differ between control and MS female mice in all three stimuli set conditions [‘female vs. male’: F (1,17) = 0.02; *p* = 0.20; ‘female vs. empty’: F (1,18) = 2.18, *p* = 0.28; ‘male vs. empty’: F (1,18) = 0.01; *p* = 0.20], social preference was greatly modified by neonatal MS. Control females showed no preference for either stimulus when they were tested with ‘female vs. male’ stimuli set ([t (7) = 0.39, *p* = 0.36]; Fig. 4A). When tested with either stimuli set condition ‘female vs. empty’ or ‘male vs. empty,’ control females preferred to spend time with another mouse when the stimulus was male [t (11) = 3.76, *p*<0.01], but no statistically significant preference was found when the stimulus was female [t (9) = 1.80, *p* = 0.10]. On the other hand, MS females clearly showed preference toward female over male stimuli when they were tested with ‘female vs. male’ stimuli set ([t (10) = 2.46, *p*<0.05]; Fig. 4B). When MS females were tested with either stimuli set condition ‘female vs. empty’ or ‘male vs. empty,’ they preferred to spend more time in the tunnel connected to a female stimulus cage rather than that connected to an empty cage [t (9) = 2.72, *p*<0.05], but no such preference was found towards the male stimulus cage [t (8) = 0.86, *p* = 0.42].

In addition, temporal changes of social preference across the 5 testing days differed towards female and male stimuli in control and MS female mice (Fig. 4C–E). When females were tested with ‘female vs. male’ stimuli set, preference towards the female stimulus increased across the 5 day testing period ([treatment: F (1,17) = 1.80, *p* = 0.20; day: F (4,68) = 6.99, *p*<0.001; treatment × day: F (4,68) = 1.78, *p* = 0.14]; Fig. 4C). When females were tested with ‘female vs. empty’ stimuli set, both MS and control females similarly showed a stable preference toward female stimulus in all 5 days of testing ([treatment: F (1,18) = 1.22, *p* = 0.29; day: F (4,72) = 0.06, *p* = 0.99; treatment × day: F (4,72) = 1.86, *p* = 0.13]; Fig. 4D). Interestingly, when presented with ‘male vs. empty,’ preference towards the male stimulus significantly decreased across the testing period ([treatment: F (1,18) = 1.80, *p* = 0.20; day: F (4,72) = 6.96, *p*<0.001; treatment × day: F (4,72) = 0.76, *p* = 0.56]; Fig. 4E).

### MS Effects on FosB Expression Following Stimulus Mice Exposure

#### Social Behaviors During Stimulus Exposure

Consistent with SIT results, cumulative duration of social investigation was significantly shorter in MS females regardless of stimulus gender ([treatment: F (1,12) = 10.52, *p*<0.01; stimuli: F (1,12) = 0.01, *p* = 0.92; treatment × stimuli: F (1,12) = 0.04, *p* = 0.84]; Table S2) during the 15 min exposure period. On the other hand, the number of stretched approaches was not significantly elevated in MS females compared to control females [treatment: F (1, 12) = 4.00, *p* = 0.07], but there was an increased number of stretched approaches when the stimulus mouse was male [stimuli: F (1,12) = 14.27, *p*<0.01; treatment × stimuli: F (1,12) = 0.71, *p* = 0.42],

#### Paraventricular Nucleus

**Figure 5:**
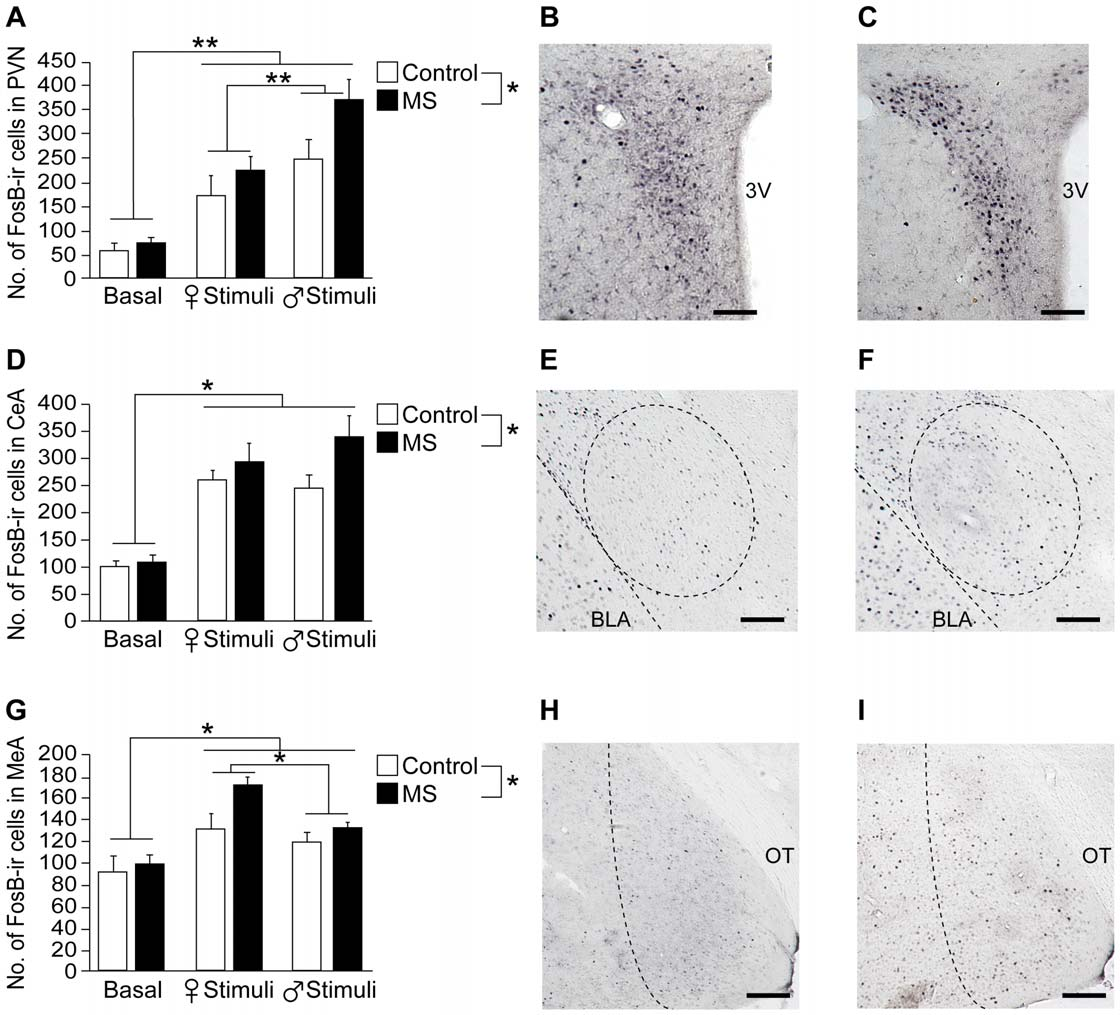
Effects of MS on FosB immunoreactivity one hour following stimulus exposure in the Paraventricular Nucleus (PVN), Central Amygdala (CeA), and Medial Amygdala (MeA). Number of FosB immunopositive cells in the (A) PVN, (D) CeA, and (G) MeA of control and MS females not exposed to a stimulus mouse (basal), exposed to an unfamiliar female stimulus mouse, or exposed to an unfamiliar male stimulus mouse for 15 min. Representative images for FosB immunoreactivity in the PVN for (B) control and (C) MS female mice exposed to a male stimulus mouse, the CeA for (E) control and (F) MS females exposed to male stimuli, and the MeA for (H) control and (I) MS female mice exposed to female stimulus mice. All data are presented as mean + SEM. Scale bar, 100 µm. 3V, third ventricle; BLA, basolateral amygdala; OT, optic tract. *, *p*<0.05; **, *p*<0.01.

It was found that there was an overall significant effect of stimulus exposure in the number of FosB positive cells in the PVN ([F (2,18) = 28.68, *p*<0.001]; Fig. 5A). Further analysis revealed that not only exposure to unfamiliar stimulus mice of each sex resulted in FosB induction over basal levels (*p*<0.01), but also exposure to male stimulus mice elicited a greater number of FosB positive cells compared to exposure to female stimulus mice (*p*<0.01). Additionally, the number of FosB immunopositive cells in the PVN was found to be significantly higher in MS females compared to control females ([F (1,18) = 5.87, *p*<0.05]; Fig. 5B for control, Fig. 5C for MS), although no significant treatment and stimuli interactions were found [F (2,18) = 1.46, *p* = 0.26].

#### Central Amygdala

FosB immunoreactivity in the CeA was also increased by exposure to either female or male stimuli mice compared to basal levels (*p*<0.05) in both MS and control females ([stimuli: F (2,18) = 35.12, *p*<0.001]; Fig. 5D). Similar to the findings in the PVN, MS females overall displayed higher number of FosB positive cells compared to control mice ([F (1,18) = 5.08, *p*<0.05]; Fig. 5E for control, Fig. 5F for MS), however, no significant treatment and stimuli interactions were found [F (2,18) = 1.71, *p* = 0.21].

#### Medial Amygdala

Increase in FosB immunoreactivity over basal levels in the MeA by stimulus exposure to each sex (*p*<0.05) was also found in the MeA of both MS and control females ([F (2,18) = 14.78, *p*<0.001]; Fig. 5G). Further analysis revealed that unlike in the PVN, number of FosB positive cells induced by female stimuli was significantly higher compared to exposure to male stimuli (*p*<0.05) regardless of treatment. As found in the PVN and CeA, MS significantly increased the number of FosB positive cells in the MeA compared to control females ([F (1,18) = 5.86, *p*<0.05]; Fig. 5H for control, Fig. 5I for MS), but no significant treatment and stimuli interaction was found [F (2,18) = 1.41, *p* = 0.27].

## Discussion

### Social Investigative Behaviors

In the present study, we provide new evidence of alterations in social behaviors by neonatal MS stress in adult female mice. We have designed SIT to examine social investigative behaviors of female mice in their home territory toward unfamiliar stimuli mice and demonstrated an elevated level of social anxiety in MS females compared to control females. When females were tested in a social context, in which they can freely approach and investigate a conspecific individual, MS females were less likely to interact with the stimulus mouse, possibly to due elevated levels of fear or apprehensive. The higher levels of social anxiety in MS females may be part of stress responses triggered by the encounter of an unfamiliar opponent, which might have then stimulated anxiogenic effects [25]. The stressful experience caused by MS during the neonatal period is known to alter the hypothalamic pituitary adrenal (HPA) axis function, the main stress response system, of adult mice and rats and results in hyperresponsiveness to stress in adulthood [3], [26], [27]. Furthermore, studies have shown that animals hyperresponsive to stressful situations are more likely to display behaviors related to social avoidance and behavioral inhibition during social encounters [28]–[31]. Interestingly, in humans, patients with social anxiety disorder, which is associated with a history of child abuse/neglect, are known to have an enhanced sensitization of the HPA axis to stress [32]. In the present study, we have also found enhanced induction of FosB immunopositive cells in the PVN, CeA, and MeA, which are regions known to be involved in the regulation of stress responses as well as social behavior, in MS females after exposure to an unfamiliar stimulus mouse. This could be an indication of an increased release of stress-related neuropeptides, such as corticotropin-releasing hormone (CRH) and arginine vasopressin (AVP). Both neuropeptides expressed in the brain are known as major regulators of HPA axis activity and have also been implicated in the expression of behavioral responses to stress [33]–[35]. Taken together, these findings collectively suggest that MS females may indeed be hyperresponsive to the stressful situation of engaging in social interaction with an unfamiliar social stimulus and thereby exhibit heightened levels of social anxiety. Future studies need to examine whether the increase in FosB immunopositive cells in the PVN, CeA, and MeA cells is indeed correlated with alterations in stress-related neuropeptides such as CRH and AVP.

### Social Preference

To our knowledge, this study demonstrates for the first time that neonatal MS may alter social preference patterns in female mice. During short-term SPT, in which a male and a female stimulus mouse were presented, MS females displayed heightened levels of social anxiety by spending less time investigating stimulus mice, similar to SIT results. Increased levels of social anxiety in the short-term SPT may have influenced social preference in MS females. As such, MS females did not display a significant preference for either female or male stimulus mice during short-term SPT like control females. On the other hand, when MS females were exposed to stimuli mice for a prolonged period in the long-term SPT, they were able to display a distinctive preference for the female mouse. Specifically, MS females consistently preferred the presence of a female stimulus mouse to the presence of a male mouse or an empty cage in the long-term SPT. On the contrary, control females showed no preference between a pair of female and male stimuli mice, which is consistent with previous studies examining odor preference in OVX female rats [36]. Also, in contrast to MS females, control females preferred to spend time with a male mouse rather than be alone, whereas such significant preferences were not found toward female stimuli. To date, effects of MS on female sexual behavior or partner preference are not well defined and our results may be one step into understanding the effects of early life stress on formation of social bonding in adulthood. In humans, fear of interacting with the opposite sex is one symptom in psychiatric disorders like social anxiety disorder [37], which is partly influenced by early adverse experiences and women are more likely to suffer from these disorders compared to men [11], [38]. Studies in rodents have shown that applying acute stress as well as corticosterone injections can inhibit partner formation in females or decrease the preference for male odors [15]–[18], suggesting a role for the HPA axis in preference for male opponents in rodents. Furthermore, it should be noted that in the present study, neonatal MS disrupted patterns of social preference for female and male opponents, but MS females did not behaviorally display phenotypes of social phobia. When control and MS females were presented with the option of spending time with another mouse or being alone with an empty cage, both groups did not display a significant preference to be alone. These results suggest that early adverse experiences may influence levels of social anxiety in adulthood, but does not necessarily induce social phobia-like behaviors. Future studies need to examine the effects of early life stress on female social behaviors that are more specifically associated with male-directed interactions, such as lordosis during sexual behavior, to better understand the behavioral phenotypes displayed towards male stimuli.

FosB induction after exposure to a stimulus mouse was found to be dependent on stimulus gender in both control and MS females in the PVN and MeA brain regions examined in the present study. Higher number of FosB immunopositive cells was induced in the PVN following the exposure to male stimuli and in the MeA following female stimuli. This region-specific effect of stimulus gender on FosB induction suggests that the neuronal circuitry and neuroendocrine responses toward an unfamiliar female and male stimuli mice might differ. Furthermore, MS stress possibly elevated the induction of FosB positive cells compared to control mice in the PVN, CeA and MeA brain regions following stimuli exposure. Greater neuronal activation in these brain regions in MS females might be associated with modifications of female social behaviors found in the present study. It is known that the PVN, CeA, and MeA express oxytocin and/or it's receptor in addition to CRH and AVP [39]–[42], which are all important regulators of social behaviors. Therefore, MS stress may differently alter the functions of these neuroendocrine correlates when female mice are exposed to female and male opponents for the first time in adulthood. Further studies need to elucidate the underlying neuronal circuitry and neuroendocrine functions associated with the stimulus gender-dependent effects on social behaviors in MS females.

### Emotional and Anxiety-Related Behaviors

MS female mice also showed altered non-social behavioral responses in unfamiliar situations such as a novel environment in OFT, which was conducted under a low light intensity condition. The stress derived from exposure to a novel environment may have contributed to inducing neophobia and suppressing exploratory behaviors in MS females, since decrease of exploratory behavior in a novel environment has been shown in female rats centrally administered with CRH [43], [44]. Furthermore, the present study demonstrated that MS affected two behavioral measurements used as indices of exploratory behaviors in OFT in opposite directions, i.e., the time spent in the center area was decreased whereas number of rears was increased by MS in female mice. Generally, these two behavioral parameters are reported to be positively correlated [45], [46]. Detailed analysis of rearing in the females revealed that although MS females exhibited more rearing, the majority of their rearing was displayed in the peripheral areas of the arena, suggesting modified coping responses to a novel environment compared to control females. Therefore, results in the present study demonstrate that MS stress may indeed alter behavioral responses of female mice as adults to stress derived from novelty and unfamiliarity in both non-social (e.g., OFT) and social (e.g., SIT) contexts. Physiological and neuroendocrine mechanisms of stress coping strategies, as a basis of behavioral changes in MS females, need to be further examined in future studies.

In contrast to greatly modified behavioral responses in OFT and SIT, MS females did not differ from control females in any behavioral parameters in LDT, a widely and reliably used test to measure animals' anxiety levels. Generally, the effect of MS on female anxiety levels measured in either LDT or elevated plus maze test, another commonly used test for anxiety-related behaviors, has remained inconsistent. Studies in mice and rats have reported that MS either increases [4], [5], [9] or decreases [47]–[49] the level of anxiety, or has no effect at all [6], [50]. Since it is known that estrogen has both anxiolytic and anxiogenic effects in female mice depending on the dose administered [10], we tested female mice as ovariectomized to simply avoid any confounding effects of circulating levels of estrogen at the time of testing with the MS effects. However, it is still possible that low endogenous levels of estradiol might be necessary to detect MS effects on anxiety levels in females since clear reduction of emotionality and anxiety levels by MS was detected in female mice tested in diestrus (low levels of estrogen) in the elevated plus maze test [8]. It is of future interest to examine whether MS may affect estrogenic regulation of anxiety-related behavior in female mice by testing ovariectomized females with different doses of estrogen treatment.

In summary, the early life period is a sensitive age in which negative life experiences can greatly alter subsequent behavioral phenotypes. We found that maternal separation stress during the neonatal developmental period can modify coping responses in a novel environment as well as disrupt social interactive behaviors to unfamiliar stimuli in adult female mice. These results suggest that early life stress experience, such as MS, can alter behavioral response patterns in situations involving nonsocial as well as social novelty in female mice.

## Supporting Information

Figure S1 **Social investigation test (SIT) apparatus.** (A) The apparatus (SOSI TYPE1) consisted of a (B) white plastic testing cage and (C) Plexiglas cylinders (Mouse Cylinder SIOT1) used to introduce the stimulus mouse. Near the bottom of the each cylinder are 28 holes that allow the experimental mouse to be exposed to olfactory cues from the stimulus mouse. For each experimental mouse, (D) duration of social investigation, (E) duration of sniffing from the corner of the cage, and (F) number of stretched approaches toward the stimulus-containing cylinder were measured as social investigative behaviors.
(TIF)

Figure S2 **Long-term social preference test (SPT) apparatus.** Top view of the long-term social preference test apparatus (AMAZENG TYPE1), which consisted of one large cage connected to two smaller cages by clear acrylic tunnels. At the end of each tunnel, wire mesh prevented physical contact between experimental and stimulus mice except for the tip of their nose. Experimental mice were housed in the large cage and the stimuli mice in the smaller cages. Infrared sensors measured the cumulative time the experimental mouse spent in each tunnel connected to the small cages housing the stimuli mice. All three cages were covered with wire metal tops with food and water compartments.
(TIF)

Table S1 **Litter effects on OFT, LDT, and short-term SPT behavioral measurements.**
(MOV)

Table S2 **MS effects on social investigative behaviors during stimulus exposure.**
(MOV)

Video S1 **Social investigation test of control and MS females tested against unfamiliar female stimuli mice.** Control females exhibited high levels of social investigation of female stimulus-containing cylinder, whereas MS females displayed numerous stretched approaches toward the stimulus-containing cylinder and low social investigation. Left, control female; right, MS female.
(DOC)

Video S2 **Social investigation test of control and MS females tested against unfamiliar male stimuli mice.** Control females, but not MS females displayed high levels of social investigation towards the male stimulus-containing cylinder. On the other hand, MS females displayed frequent stretched approaches. Left, control female; right, MS female.
(DOC)
