# Supplementary figures and images for: Long-Lasting Consequences of Neonatal Maternal Separation on Social Behaviors in Ovariectomized Female Mice
Source: PLoS One. 2012 Mar 7;7(3):e33028. doi: 10.1371/journal.pone.0033028 (PMC3296763; doi:10.1371/journal.pone.0033028)

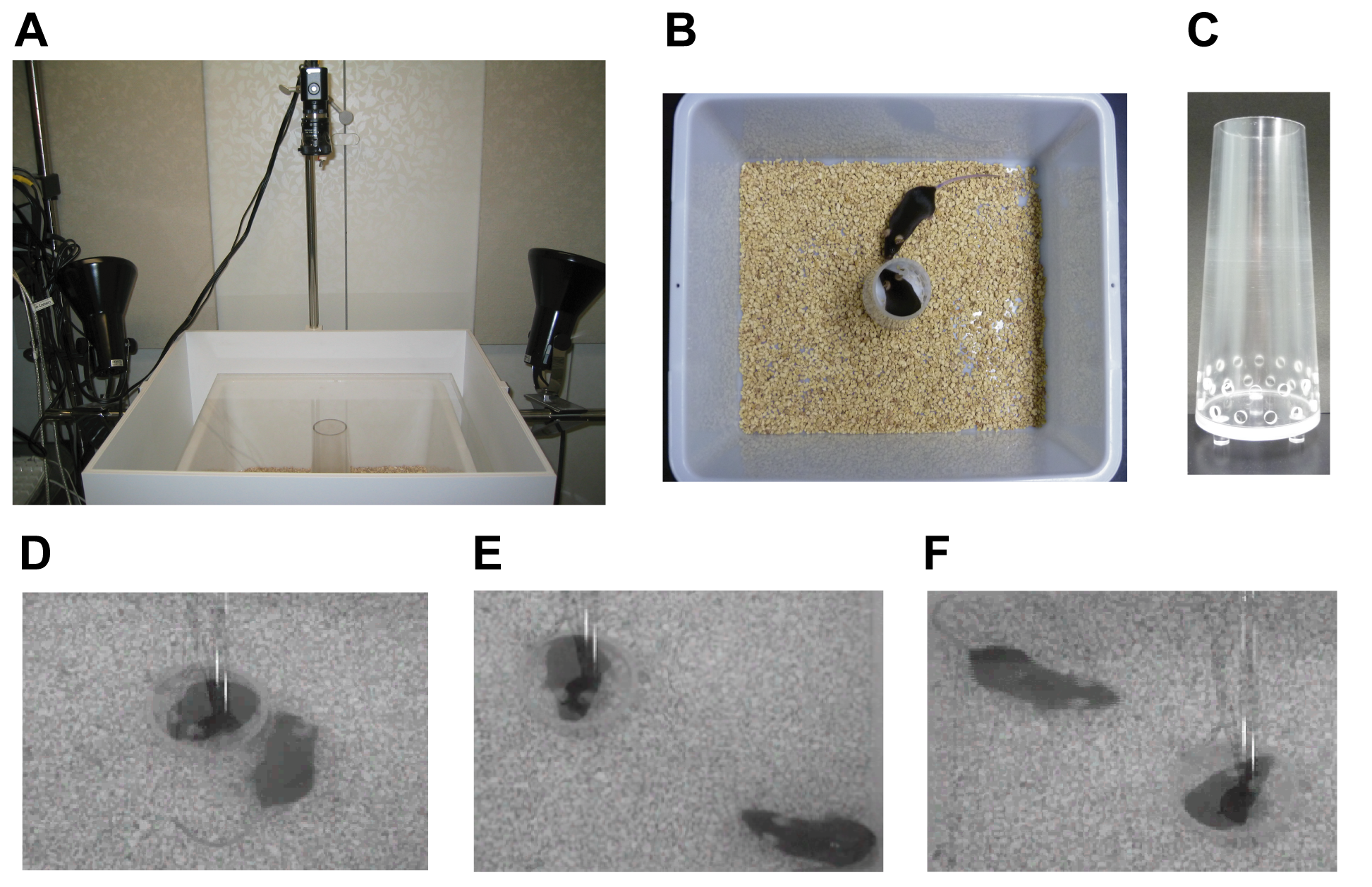

Supplement: Figure S1 — Social investigation test (SIT) apparatus. (A) The apparatus (SOSI TYPE1) consisted of a (B) white plastic testing cage and (C) Plexiglas cylinders (Mouse Cylinder SIOT1) used to introduce the stimulus mouse. Near the bottom of the each cylinder are 28 holes that allow the experimental mouse to be exposed to olfactory cues from the stimulus mouse. For each experimental mouse, (D) duration of social investigation, (E) duration of sniffing from the corner of the cage, and (F) number of stretched approaches toward the stimulus-containing cylinder were measured as social investigative behaviors. (TIF) [file pone.0033028.s001.tif]

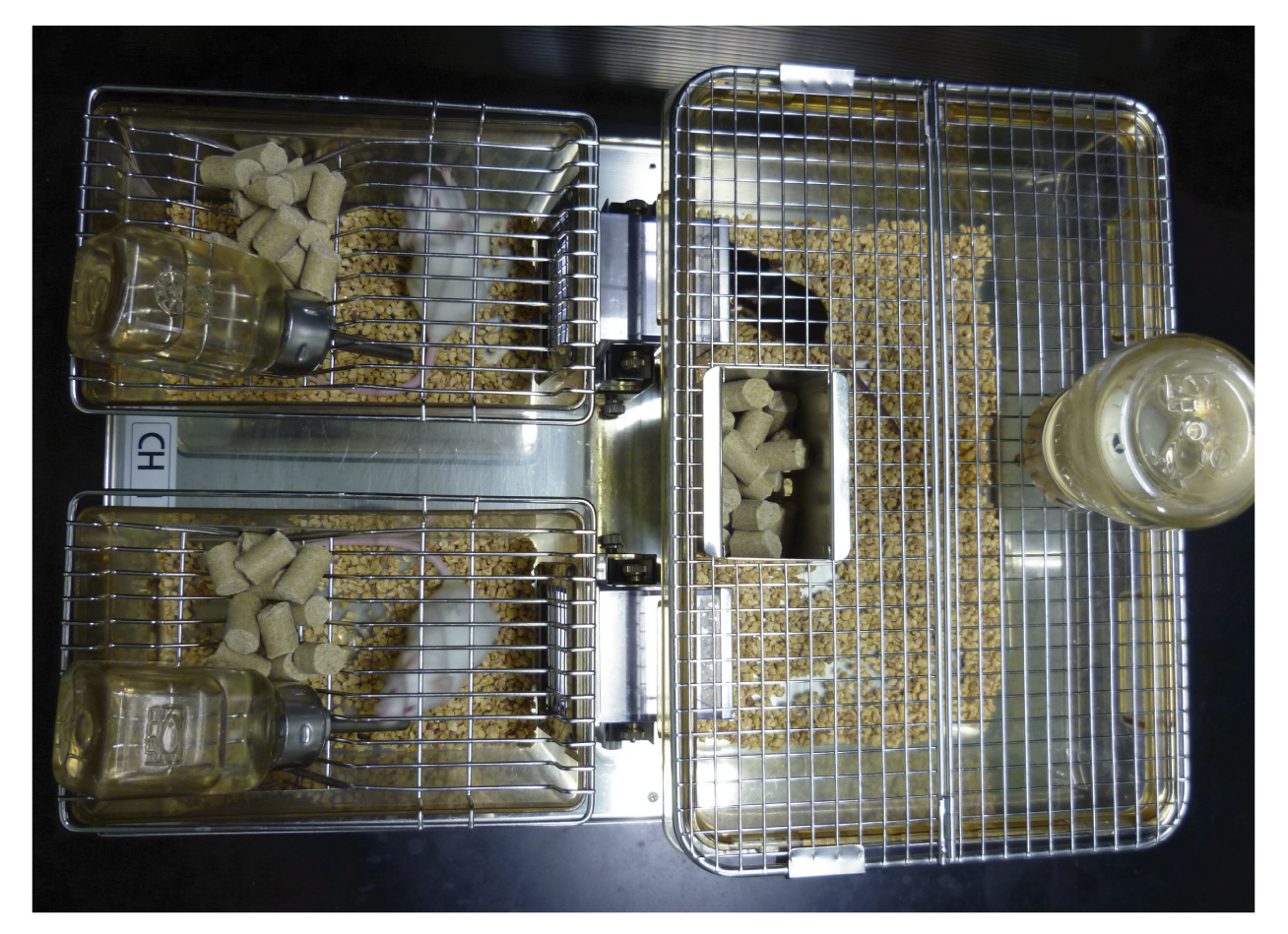

Supplement: Figure S2 — Long-term social preference test (SPT) apparatus. Top view of the long-term social preference test apparatus (AMAZENG TYPE1), which consisted of one large cage connected to two smaller cages by clear acrylic tunnels. At the end of each tunnel, wire mesh prevented physical contact between experimental and stimulus mice except for the tip of their nose. Experimental mice were housed in the large cage and the stimuli mice in the smaller cages. Infrared sensors measured the cumulative time the experimental mouse spent in each tunnel connected to the small cages housing the stimuli mice. All three cages were covered with wire metal tops with food and water compartments. (TIF) [file pone.0033028.s002.tif]
